# A Multi-Gene Signature Associated with 1-Year Survival in Patients with Stage I Liver Cancer: Integration of Preclinical and TCGA Data

**DOI:** 10.3390/cimb48020136

**Published:** 2026-01-27

**Authors:** Ritam Adhikari, Bhaskar V. S. Kallakury, Chiranjeev Dash, Rabindra Roy

**Affiliations:** 1 The Quarry Lane School, 6363 Tassajara Rd., Dublin, CA 94568, USA; ra1314@georgetown.edu; 2 Department of Oncology, Lombardi Comprehensive Cancer Center, Georgetown University Medical Center, Washington, DC 20057, USA; cd422@georgetown.edu; 3 Department of Pathology, Lombardi Comprehensive Cancer Center, Georgetown University Medical Center, Washington, DC 20057, USA; kallakub@georgetown.edu

**Keywords:** microarray, liver cancer, machine learning, IPA, Stage I, prognostic panel, NGS

## Abstract

Approximately 50% of individuals diagnosed with Stage I liver cancer live beyond four years; however, a small subset of Stage I patients die within the first year. A prognostic biomarker panel that can identify high-risk Stage I patients may be extremely valuable. In this study, we used the Long–Evans Cinnamon (LEC) rat model of Wilson’s Disease and hepatocellular carcinoma (HCC), along with data from The Cancer Genome Atlas (TCGA) human database, to create a novel biomarker panel. We generated and analyzed a rat microarray gene expression profile by comparing liver tumor tissues with adjacent normal tissues from the same animals, covering approximately 30,000 genes. The microarray results were translated into a five-gene panel associated with 1-year survival in Stage I liver cancer patients based on TCGA data, in combination with machine learning and bioinformatics approaches. The panel was internally validated following the “REporting recommendations for Tumor MARKer prognostic studies (REMARK)” guidelines. With no existing Stage-I-specific prognostic tools, a biomarker panel associated with 1-year survival in patients with Stage I liver cancer is a potential candidate for rigorous external validation.

## 1. Introduction

Since 1980, the incidence of liver cancer has tripled, and its death rate has doubled [1,2]. Factors such as alcohol consumption, metabolic dysfunction-associated liver disease (MASLD), hepatitis virus infections, and genetic disorders involving copper or iron overload contribute to liver cancer, one of the leading causes of cancer-related deaths worldwide [3,4]. Although these etiologic factors differ, they converge on a common pathogenic mechanism: they induce chronic hepatic inflammation and fibrosis, which are major drivers of hepatocarcinogenesis [5,6,7].

The prognosis of patients with liver cancer depends on the cancer stage. Approximately half of the individuals diagnosed with Stage I disease have a relatively favorable prognosis and live beyond four years (https://www.cancerresearchuk.org/about-cancer/liver-cancer/survival; https://www.hepb.org/research-and-programs/liver/staging-of-liver-cancer/survival-rates, both accessed on 30 December 2025). Patients diagnosed with Stage I liver cancer are treated with the standard of care (SOC) (liver resection, transplantation, or ablation) [8,9]. However, a small subset of patients with Stage I disease die within the first year [10]. Developing a prognostic biomarker panel to identify high-risk Stage I patients could be clinically beneficial for identifying those who may benefit from innovative treatments, including newer therapies. Currently, no prognostic biomarker panel specifically focused on Stage I liver cancer is available.

The Long–Evans Cinnamon (LEC) rat model, which mimics Wilson’s Disease (WD), naturally accumulates copper in the liver due to a 900 bp deletion in the rat Atp7b gene, leading to liver injury and hepatitis, which are common initiating events in hepatocellular carcinoma (HCC) [11,12,13,14]. The LEC model is important for understanding and treating fibrosis- and inflammation-mediated HCC.

In this study, we hypothesized that differentially expressed genes between liver cancer and adjacent normal tissues may provide novel insights into cancer sustenance and progression, potentially revealing new prognostic signatures for the disease. We generated and analyzed a rat microarray gene expression profile by comparing liver tumors and adjacent normal tissues from the same LEC rats, covering approximately 30,000 genes. Using an array of machine learning pipelines and the Cancer Genome Atlas (TCGA) database for liver cancer, we translated the rat microarray results into a five-gene signature panel associated with the 1-year prognosis of Stage I liver cancer.

## 2. Materials and Methods

### 2.1. Animal and Liver Tissues

LEC rats were maintained under standard conditions following the IACUC-approved protocol (#07-065) [15]. Liver tissues were collected from three LEC rats at 84 weeks, paraffin-embedded, and stained with hematoxylin and eosin (H and E). Adjacent normal (late chronic hepatitis, tumor-adjacent normal) and tumor tissue sections, both collected at 84 weeks, were identified after histopathological review by a pathologist who was board-certified in hematopathology, anatomic pathology, and clinical pathology, as described previously [15].

### 2.2. Total RNA Extraction and Gene Expression Microarray

We performed a gene expression microarray analysis using total RNA to identify differences in gene expression between tumor and adjacent normal tissues. Total RNA was isolated from the 84-week adjacent normal and 84-week tumor LEC liver tissues using the RNAeasy (Qiagen, Valencia, CA, USA) kit, according to the manufacturer’s instructions. Total RNA extracted from the tissues was hybridized to the Affymetrix GeneChip Rat Genome 230 2.0 Array (Affymetrix Inc., Santa Clara, CA, USA) and probed for 30,000 rat transcripts. Raw data were background-corrected, normalized, and RMA-summarized. Differential expression analysis between 84-week tumor and 84-week adjacent normal samples was performed using Biometric Research Branch (BRB)-ArrayTools, which applies a Random Variance Model (RVM). Fold changes between the 84-week tumor and 84-week adjacent normal tissues, as well as the associated *p*-values, were calculated to identify statistically significant (*p*-value ≤ 0.05) differentially expressed genes between the two groups. In addition, a fold change cutoff of ±2 and an FDR < 0.25 cutoff were used to select genes for analysis following GSEA guidelines [16].

### 2.3. Visualization of Gene Expression Data with Volcano Plot

A volcano plot was generated to visualize the significant gene expression changes between 84-week tumors and 84-week adjacent normal tissues, with a cutoff of +/− 2-fold. The Python seaborn package (.sns) was used for the volcano plot to visualize changes between 84-week tumor and 84-week adjacent normal samples. Python 3.12.12 (Google colaboratory) was used for this and all other downstream analyses.

### 2.4. Agglomerative Hierarchical Clustering

Hierarchical clustering was employed to identify potential additional clusters within the upregulated and downregulated groups between the 84-week tumor and 84-week adjacent normal tissues [17] using the Python package schlearn (.sch).

### 2.5. K-Means Clustering and Elbow Method

We determined the optimal number of clusters using the “elbow” method within the upregulated and downregulated groups by comparing 84-week tumor and 84-week adjacent normal tissues. K-means clustering was then employed to cluster and graphically represent the data in a 3D scatter plot, rather than a dendrogram [18].

### 2.6. Ingenuity Pathway Analysis for Each Cluster

Each cluster obtained from the K-means clustering analysis within the over- and under-expressed groups was subjected to pathway analysis using QIAGEN Ingenuity Pathway Analysis (QIAGEN IPA) to understand the interplay between biological pathways. In addition, results with −log(*p*) > 1.3, a z-score cutoff ≥ 2 for pathway activation, and a z-score cutoff ≤ −2 for pathway inhibition were considered significant.

### 2.7. Human Protein Atlas (HPA), OncoLnc, and Kaplan–Meier (KM) Plotter for Potential Prognostic Biomarkers

The top ten genes from the clusters obtained from K-means clustering analysis of the over- and under-expressed groups were investigated for their prognostic potential using three web-based tools—Human Protein Atlas (HPA), OncoLnc, and KM plotter-based log-rank test (Mantel–Cox Test), with *p*-value < 0.05 (HPA and KM plotter)—as well as hazard ratios and the available literature.

### 2.8. Analysis of TCGA Stage I Liver Cancer Patient Data

We extracted RNA-seq TPM expression data from HPA for the top candidate genes with potential prognostic value for Stage I liver cancer patients, along with associated clinical metadata, including survival time, survival status, tumor stage, age, and sex, from the TCGA liver cancer dataset.

### 2.9. One-Year Survival Prediction by Age and Sex for Stage I Liver Cancer

We fit Cox proportional hazards models to assess whether age and sex predict 1-year survival. First, we used lifelines’ CoxPHFitter on age and sex. In parallel, we trained a scikit-survival pipeline—StandardScaler for age and OneHotEncoder(drop = “if_binary”) for sex, feeding CoxPHSurvivalAnalysis—with a 70/30 train–test split stratified by the event indicator. Discrimination at 365 days was evaluated with time-dependent AUC (cumulative_dynamic_auc), and overall ranking with the IPCW C-index—inverse-probability-of-censoring-weighted concordance—which adjusts for right-censoring by reweighting pairs using the estimated censoring distribution.

### 2.10. Time-Dependent Prognostic Significance Analysis of Individual Genes for Stage I Liver Cancer

Univariate Cox proportional hazards modeling was performed separately for 1-, 2-, 3-, and 5-year time points for each gene using survival data (days and status) and gene expression levels. Patients were censored if they were alive before the defined time point but had no follow-up information beyond that point.

We first identified optimal thresholds to group patients into “High” and “Low” expression groups using the lifelines.statistics.logrank_test package. Gene expression quantile thresholds from the 20th to 80th percentile were tested and compared for their impact on survival distributions. The threshold yielding the smallest *p*-value (log-rank test) was selected as the optimal cutoff.

For each gene and time point, a univariate Cox proportional hazards model was fitted using the CoxPHFitter module in Python lifelines to estimate the hazard ratio (HR), 95% confidence interval (CI), *p*-value, and time-dependent area under the curve (AUC). Kaplan–Meier survival curves were generated (Kaplan–Meier Fitter).

### 2.11. Ranking Top Genes for Predicting 1-, 2-, 3-, and 5-Year Survival

To identify the top-performing prognostic genes at each time point (1, 2, 3, and 5 years), we ranked the genes with the highest AUC values derived from the univariate Cox models and then selected the top five. With the selected genes, we constructed four time-specific five-gene signature panels, referred to as Panel-A, Panel-B, Panel-C, and Panel-Ds, for downstream analysis.

### 2.12. Time-Dependent Multivariate LASSO Regression for Survival and Gene Selection

We applied LASSO regression (sklearn.linear_model.LassoCV) to standardized gene expression values (sklearn.preprocessing.StandardScaler) to construct a separate multivariate risk score for each of the four time-specific panels: Panel-A, Panel-B, Panel-C, and Panel-D.

### 2.13. Multivariate Cox Regression Survival Analysis (Time-Dependent AUC)

The four prognostic panels (Panel-A, Panel-B, Panel-C, and Panel-D) were used to train a separate multivariate survival model using the Cox proportional hazards model (CoxPHSurvival Analysis from scikit-survival), with the corresponding five genes included as covariates.

The performance of each model was evaluated using cumulative dynamic time-dependent AUC calculations (cumulative_dynamic_auc, scikit-survival) at one, two, three, and five years. This method accounts for right-censored data and compares risk score predictions with survival status at specified future time points. AUC values with 95% and 80% CIs were calculated for each model across all evaluation years and visualized using matplotlib.pyplot to compare the cross-time point prognostic performance.

### 2.14. Classical Receiver Operating Characteristic (ROC) Analysis

ROC analysis was performed using the roc_curve and auc functions from the sklearn.metrics module. Cox proportional hazards models (CoxPHSurvivalAnalysis, scikit-survival) were trained with each of the four prognostic panels (Panel-A, Panel-B, Panel-C, and Panel-D) at each evaluation year (1, 2, 3, and 5 years). The models’ predicted risk scores were evaluated against the derived binary outcome labels.

Patients were labeled as positive if they died in or before the specified evaluation year and negative if they were alive beyond that point; they were censored if they were alive before the time point but lacked follow-up information beyond that point. ROC curves were constructed, and the AUC was calculated to assess the discriminative ability of the risk scores at each evaluation time point.

### 2.15. Generation of Kaplan–Meier Survival Curves by Comparing Optimized Risk Groups Across Four Panels

Multivariable Cox proportional hazards models were fitted using the four prognostic gene panels to predict survival at 1, 2, 3, and 5 years (CoxPHSurvival Analysis, sksurv.linear_model). For each panel, patients were stratified into “High” and “Low” risk groups based on an optimal cutoff determined by minimizing the log-rank *p*-value across the 20th to 80th percentile of the predicted risk score distribution (logrank_test, lifelines.statistics).

For each risk stratification, we further calculated the classification performance metrics by identifying thresholds that achieved 95% and 99% specificity in predicting events at each time point. The corresponding sensitivity values were computed using the confusionmatrix function from sklearn.metrics.

### 2.16. Calibration Curves for Multivariable Cox Survival Models Constructed Using the Four Prognostic Panels at 1-, 2-, 3-, and 5-Year Time Points

To assess the calibration of our multivariable Cox survival models, we predicted individual survival probabilities at future time points (1, 2, 3, and 5 years) using model-derived survival functions (predict_survival_function, sksurv.linear_model). Each model corresponds to one of the four prognostic panels developed previously.

The patients were grouped into five bins based on the quantiles of the predicted survival probability. For each bin, we computed the average predicted survival and compared it with the observed survival fraction to generate the calibration curves. Observed survival was defined as 1 for patients who were alive beyond the evaluation time point and 0 for those who experienced an event (death) at or before that time. Patients censored before the evaluation time point were excluded from the analysis to ensure accuracy of the results. The calibration curve function from sklearn.calibration was used to compute the observed versus predicted survival values for each bin.

### 2.17. Harrell’s Concordance Index (C-Index) for Cox Model Predictions Across Four Prognostic Panels

Multivariable Cox proportional hazards models were trained using the four prognostic panels (Panel-A, Panel-B, Panel-C, and Panel-D). Model performance was evaluated at 1, 2, 3, and 5 years by computing the concordance index (C-index) using the concordance_index_censored function in the sksurv.metrics module.

### 2.18. Bootstrapping for Risk Group Hazard Ratios

To estimate the robustness of hazard ratios (HRs) and the corresponding 95% confidence intervals (CIs) for the four prognostic panels at each evaluation year (1, 2, 3, and 5 years), we performed 100 bootstrap resamples using resamples from sklearn.utils. For each resample, a multivariable Cox proportional hazards model was fitted to calculate risk scores based on the five genes in the corresponding panel.

Simulated patients were grouped into “High” and “Low” risk groups using an optimal risk score threshold identified from the minimum log-rank *p*-value measured across quantiles from the 20th to 80th percentile (logrank_test, lifelines.statistics). A univariate Cox model with the binary risk group as a covariate was then fitted using CoxPHFitter (lifelines) to estimate the hazard ratio (HR).

The 95% confidence intervals for the hazard ratios (HRs) were computed using log-transformed HR distributions of the 2.5th and 97.5th percentiles across bootstrap iterations.

### 2.19. Hazard Ratios (HRs) and Concordance Indices (C-Indices) from 5-Fold Cross-Validation

A multivariable Cox proportional hazards model (CoxPHSurvivalAnalysis, scikit-survival) was trained using 5-fold cross-validation for each of the four prognostic gene panels for each evaluation year (1, 2, 3, or 5 years).

Within each training fold, an optimal risk score cutoff was identified by minimizing the log-rank *p*-value, as described previously. This threshold was then applied to both the training and test sets to assign patients to “High”- or “Low”-risk groups. Hazard ratios (HRs) were estimated from the test set using univariate Cox models (CoxPHFitter, lifelines) with the binary risk group as the sole covariate.

To evaluate discriminative performance, the concordance index (C-index) was computed on the test set using continuous risk scores (concordance_index_censored, sksurv.metrics). The mean HRs, confidence intervals, and C-index statistics were aggregated across valid folds to assess the prognostic accuracy and robustness of each panel at different time points.

### 2.20. Brier Score Evaluation for Survival Predictions

Brier scores were computed to assess the accuracy and calibration of the survival probability estimates from each gene panel when compared to actual outcomes at 1, 2, 3, and 5 years [19]. For each panel, the top five genes were binarized using their respective optimal cutoff values, and a Cox proportional hazards model was trained using CoxPHSurvival Analysis from scikit-survival.

Predicted survival probabilities were obtained from the model’s estimated survival functions (predict_survival_function) and interpolated at each evaluation time point using the Brier score function from sksurv. Metrics were used to compute time-dependent Brier scores, which quantify the mean squared difference between the predicted survival probability and actual outcome (alive vs. event) at a given time.

## 3. Results

### 3.1. Overview of Study Design and Workflow

**Figure 1:**
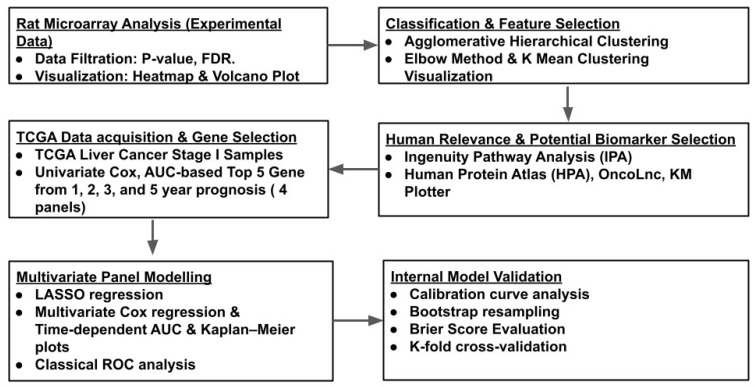
Workflow to develop and internally validate a five-gene prognostic panel derived from an in-house rat model and human TCGA data for Stage I liver cancer patients.

In this study, we aimed to develop a biomarker panel associated with 1-year survival in patients with Stage I liver cancer. As illustrated in Figure 1, we generated and analyzed differential gene expression profiles between tumor and adjacent normal liver tissues from the same animals, namely, Long–Evans Cinnamon (LEC) rats—a model of WD and HCC. Human orthologs of the top candidate genes were evaluated using Stage I liver cancer data from The Cancer Genome Atlas (TCGA). We applied a machine learning-based workflow that included unsupervised clustering, univariate and multivariate Cox proportional hazards modeling, and LASSO-based feature selection. The resulting panels were validated internally through bootstrap resampling, Brier scores, and stratified five-fold cross-validation.

### 3.2. Transcriptomic Analysis of 84-Week LEC Rat Liver Tumor and Adjacent Normal Tissues

**Figure 2:**
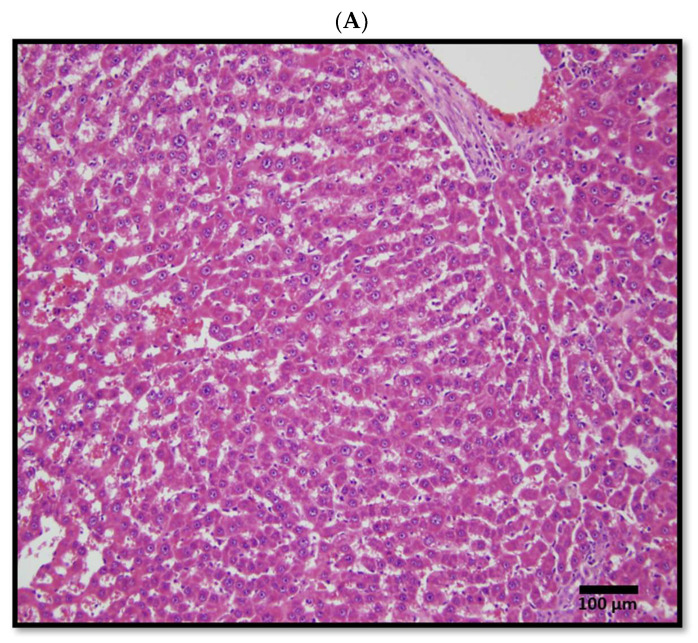

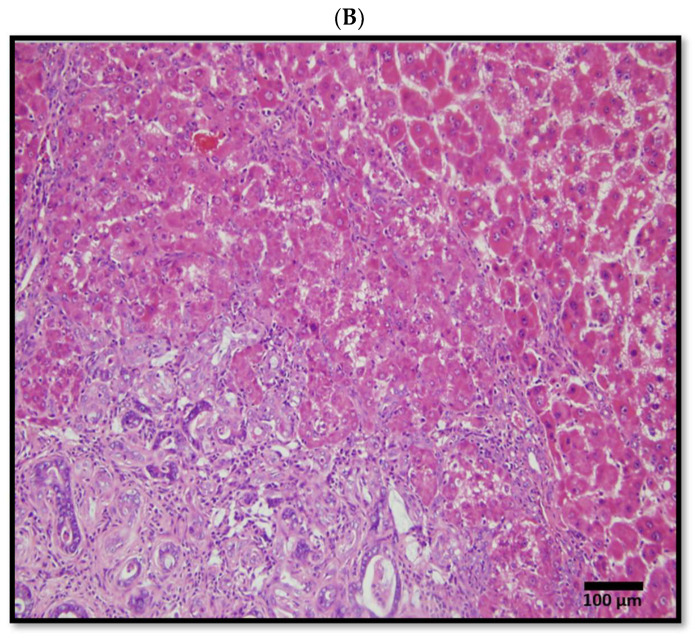
Tissues from an 84-week-old animal. Adjacent normal tissue (**A**) and tumor tissue (**B**) samples were stained with hematoxylin and eosin (H&E). Scale bar: 100 µm.

Representative hematoxylin and eosin (H&E)-stained images of 84-week tumor and 84-week adjacent normal samples are shown in Figure 2. A board-certified pathologist microscopically identified all tumors and adjacent normal tissues using H&E-stained images. Total RNA was extracted from tumor and adjacent normal tissues from three 84-week LEC rats for Affymetrix GeneChip Rat Genome 230 2.0 Array analysis (Affymetrix Inc., Santa Clara, CA, USA).

### 3.3. Volcano Plot and Significantly Expressed Genes in 84-Week Tumor vs. 84-Week Adjacent Normal Tissues

**Figure 3:**
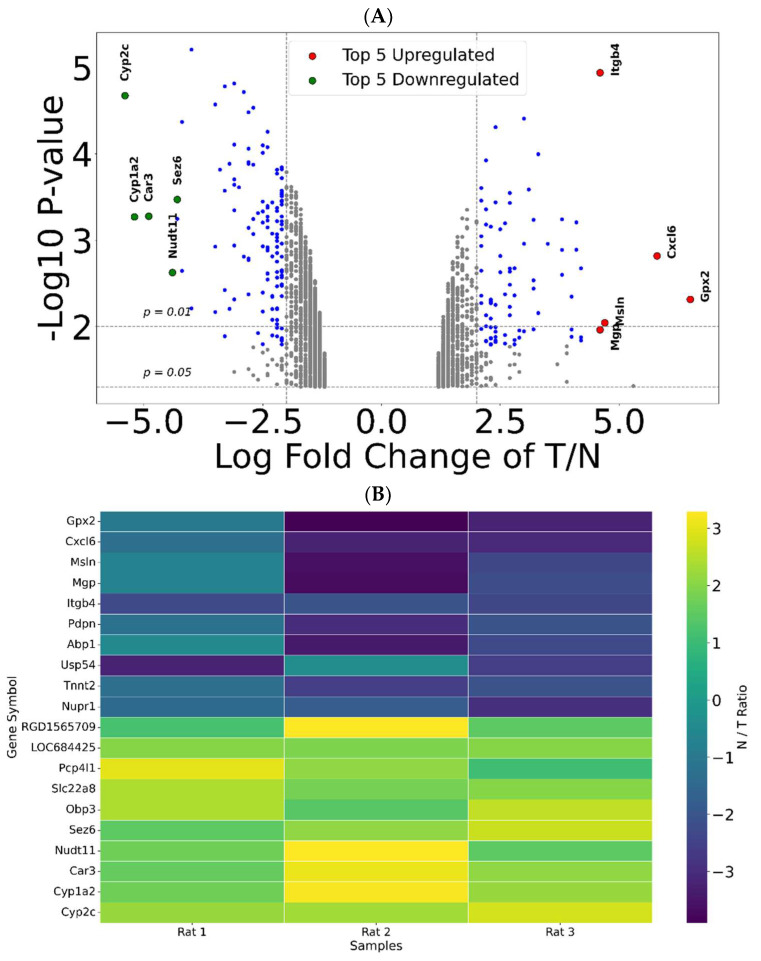
Bird’s-eye view of gene expression changes. A volcano plot (**A**) is used to visualize the significant gene changes (blue and red). A negative log2 fold change (left side) indicates downregulated genes in the tumor, while a positive log2 fold change (right side) indicates upregulated genes in tumor tissue. A total of 204 statistically significant gene expression differences were detected. All downstream work used statistically significant data. A heatmap (**B**) of the top statistically significant genes among the three rats is shown. Our results suggest that gene expression changes were similar among the three rats.

We then curated and visualized the total number of genes using a volcano plot, which revealed significant changes in their expression between the 84-week tumor and 84-week adjacent normal samples. Since both tissues were collected from the same rat and were adjacent, genes with significant changes in expression may be essential for forming and/or sustaining tumors (Figure 3A,B). The volcano plot was generated using data filtered for gene expression changes of ±2-fold (Figure 3A). The results also suggested that gene expression changes were similar among the three rats (Figure 3B). The 204 affected genes were divided into two groups: upregulated and downregulated genes. The False Discovery Rate (FDR) was less than 0.25, suggesting that among the 204 genes detected as having significant changes in expression, false identifications occurred at a rate of less than 25%. The criterion FDR < 0.25 aligns with GSEA guidelines [16] and was selected for exploratory purposes. These potential candidates were subsequently evaluated with TCGA data, reducing the risk of false discovery. Although 204 may seem to be a small number of genes, it is important to note that we applied a stringent ±2-fold cutoff and compared tumor tissue with adjacent normal tissue rather than using a separate control rat. A higher fold change reduces the background signal and increases the relevance of transcriptomic analysis.

### 3.4. Agglomerative Hierarchical Clustering and Dendrograms of Downregulated and Upregulated Genes

The volcano plot suggested that the genes that were significantly different between the 84-week tumor and 84-week adjacent normal samples could be divided into two groups: upregulated and downregulated genes. We separately tested each group using Agglomerative Hierarchical Clustering analysis and obtained two distinct clusters within both the upregulated and downregulated dendrograms (Figure S1A,B). We clustered up- and downregulated genes separately to enable clearer sub-clustering and avoid obscuring biologically meaningful patterns.

### 3.5. K-Means Clustering, Elbow Method, and Pathway Analysis

**Figure 4:**
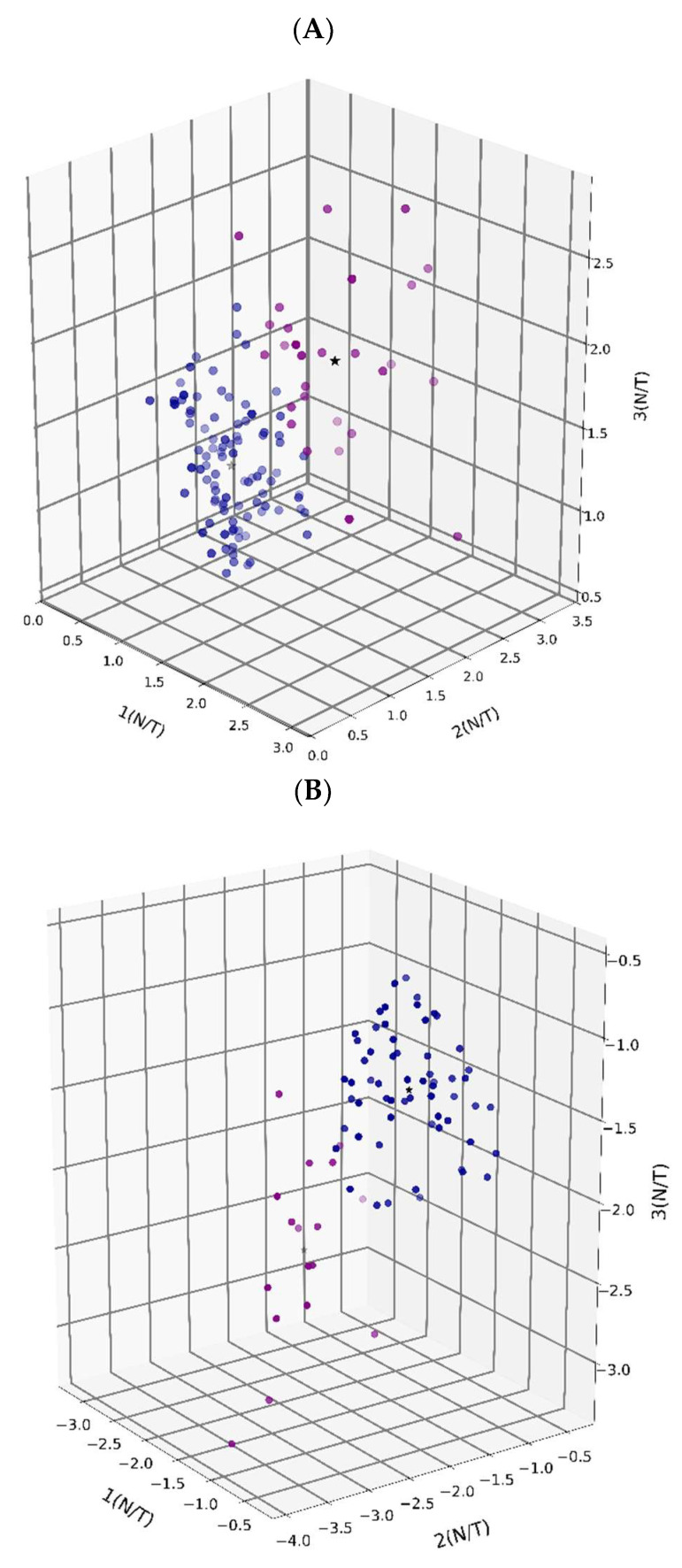
K-means clustering and elbow method. K-means clustering helps to visualize the clusters among the downregulated and upregulated genes. The elbow method suggested two optimal clusters for both downregulatedand upregulated genes, and the results are consistent with those from hierarchical clustering methods. K-means clustering helped us cluster genes within the downregulated (**A**) and upregulated genes (**B**). Blue and red denote two different clusters, and star denotes the centroid.

The elbow method also indicated two distinct clusters within the upregulated and downregulated genes. K-means clustering analysis further grouped the genes into two subclusters for each of the upregulated and downregulated groups (Figure 4A,B). The Ingenuity Pathway Analysis (IPA) tool (Figure S2) identified six pathways previously associated with human HCC, strengthening confidence in the biological relevance of our findings. To strengthen this observation, the candidate genes were subsequently compared with human databases for their prognostic relevance, providing further support beyond the IPA.

### 3.6. Survival-Associated Genes from Human Protein Atlas (HPA), OncoLnc, and Kaplan–Meier (KM) Plotter

We translated rat genes to their human orthologs using the online tool Database for Annotation, Visualization, and Integrated Discovery (DAVID) and tested the top 10 genes by effect size (Table S1) in each cluster to test their relevance for liver cancer survival. We used the web-based tools HPA, OncoLnc, and KM plotter for survival probability using TCGA liver cancer data. We selected 11 genes (hazard ratio < 0.7, hazard ratio > 1.5, and log-rank *p*-value < 0.05) with prognostic potential in liver cancer (Table S2). A literature review indicated that 8 out of the 11 genes have been previously reported by different groups to be potential biomarkers in liver cancer. *USP54* has also been used as part of a prognostic panel for HCC. No research was found related to *MGP, GK,* and *PAQR9* proteins as prognostic markers for HCC. However, *MGP* has been shown to be a possible prognostic marker for breast cancer, *PAQR9* is linked to lung cancer prognosis, and *GK* is linked to esophageal carcinoma prognosis [20,21,22,23,24,25,26,27,28,29,30]. To address the unmet clinical need to identify high-risk patients with Stage I liver cancer, we next assessed the prognostic relevance of individual genes and the collective impact of multi-gene panels on 1-year survival prediction.

### 3.7. Stage I Liver Cancer Patient Information at TCGA

We downloaded clinical and genomic data for patients with Stage I liver cancer from The Cancer Genome Atlas (TCGA) database. Although Stage I patients are generally expected to have favorable outcomes and are typically managed with standard-of-care therapies, early mortality occurs in a small but significant subset. In our curated dataset, 17 of 149 patients with Stage I disease (11.4%) died within the first year of diagnosis (Table 1). The TCGA dataset included more male than female patients, reflecting the general trend that males are more likely to be diagnosed with HCC than females [31]. The median age of 59 years in the TCGA dataset closely aligns with the known median age at HCC diagnosis in the general population [32]. The primary objective of our downstream analysis was to identify the transcriptomic features that distinguish high-risk patients from those with prolonged survival.

### 3.8. Association of Age and Sex with 1-Year Survival

Age and sex were not associated with 1-year survival. In lifelines, age HR ≈ 1.010 (*p* = 0.207) and sex (male from female) HR ≈ 0.837 (*p* = 0.368) were not significant. The scikit-survival AUC for 1 year was 0.546 (age plus sex) vs. 0.542 (age only), and C-index (test) = 0.436, indicating minimal significance of sex or age.

### 3.9. Prognostic Significance of Individual Genes for 1-, 2-, 3-, and 5-Year Survival for Stage I Liver Cancer

We evaluated the prognostic potential of 11 genes in Stage I liver cancer patients using univariate Cox proportional hazards analysis at four clinically relevant time points: 1, 2, 3, and 5 years (Table S3). Kaplan–Meier survival curves and corresponding Wald test *p*-values for these genes suggest how their expression correlates with survival probability, as shown in Figure S3. Time-dependent area under the curve (AUC) analysis revealed varying prognostic performance across genes and time points (Table 2A). AUC values below 0.5 for individual genes reflect inverse or weak associations with the outcome. These effects are supported by the corresponding hazard ratios reported (Table 2A and Table S3). These candidate genes’ AUCs were treated as a potential feature and, thus, based on AUC ranking, we constructed four gene panels, referred to as Panel-A (*ANXA2, AOC1, GK, PAQR9, USP54*), Panel-B (*ANXA2, AOC1, GK, ITGB4, USP54*), Panel-C (*ANXA2, AOC1, CD24, ITGB4, USP54*), and Panel-D (*ANXA2, FXYD3, ITGB4, PAQR9, USP54*), each consisting of the top five genes for the respective time points (Table 2B). For downstream multivariate analysis, we built sixteen Cox models by applying each of the four gene panels across the four time points.

### 3.10. Multivariate LASSO Regression for 1-, 2-, 3-, and 5-Year Survival to Evaluate Whether Any Gene in a Given Panel Could Be Excluded

We tested whether any single gene within each panel was sufficient to predict survival using LASSO-regularized Cox regression. In all cases, LASSO did not retain any individual genes, indicating that no gene is a strong independent predictor. For example, while *GK* showed moderate prognostic performance with an AUC of 0.659 at the 1-year time point in the univariate analysis, it was not selected by LASSO. As an alternative, we used the unweighted average expression of all five genes in each panel to calculate risk scores and, in some multi-gene panels, achieved AUCs greater than 0.65 (Table 3). Notably, several FDA-approved clinical assays have AUCs in this range, so these observations are encouraging [33,34]. These findings suggest that although no single gene was dominant, the cumulative signal of the multi-gene panel may help predict high-risk patients.

### 3.11. Multivariate Cox Modeling with Gene Panels

**Figure 5:**
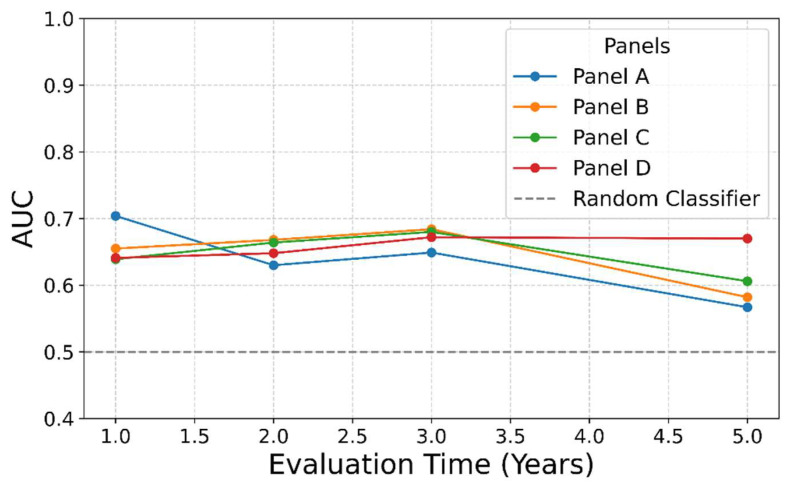
Multivariate Cox modeling shows that gene panels obtain better AUC than any single gene. At each time point, at least one model showed a better AUC than any single gene (Table 2), suggesting the strength of combining genes in the panel.

To assess the added value of combining genes into multi-gene panels, we evaluated all four prognostic gene panels using multivariate Cox regression with binary predictors, followed by time-dependent AUC analysis at 1-, 2-, 3-, and 5-year time points (Figure 5 and Table 4). The 95% and 80% confidence intervals for the multi-gene panels are reported. As anticipated, some confidence intervals at the early time point with fewer observed events do include 0.5, reflecting statistical uncertainty due to the limited number of events, which does not imply that the point estimate is equivalent to chance. This approach complements our earlier LASSO analysis, in which no single gene was selected as an independent predictor across the panels. In contrast, the multi-gene panel models demonstrated improved prognostic performance; at each evaluation time point, at least one panel achieved a higher AUC than any of its genes alone (Table 2), indicating the added value of the combined gene signal. These results are consistent with our univariate findings and reinforce the importance of a multi-gene-based strategy for identifying high-risk patients with Stage I liver cancer.

### 3.12. Classical Receiver Operating Characteristic (ROC) Curves Based on Risk Scores from Multivariate Cox Models

**Figure 6:**
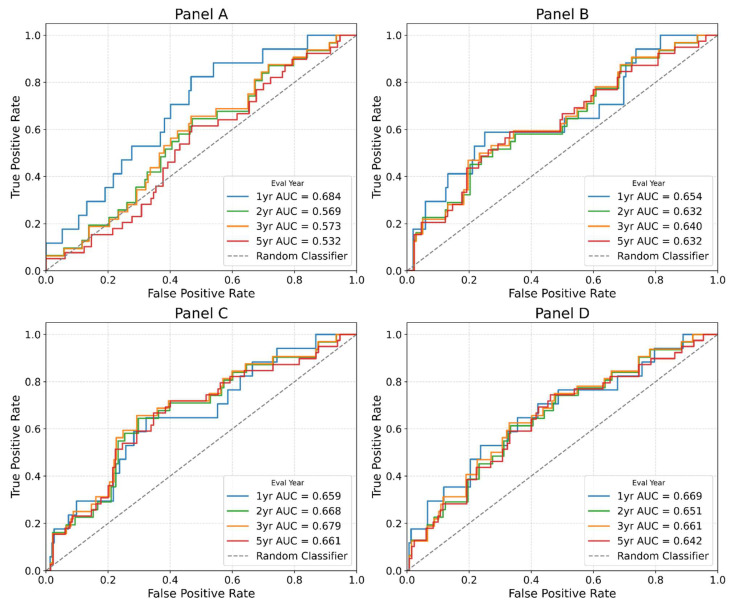
Classical ROC curve with each gene panel to evaluate AUC. To further establish the utility of the multi-gene panel, we performed classical ROC curve analysis and obtained similar AUC values for the top panels.

To evaluate the performance of each multi-gene panel, we also generated classical ROC curves based on risk scores from multivariate Cox models (Figure 6). Panel-A had an AUC of 0.684 at 1 year. Panel-D maintained similar AUCs across time points, ranging from 0.642 to 0.669.

### 3.13. Kaplan–Meier Plots and Sensitivity/Specificity Analysis

To visualize the prognostic utility of the panels (Panel-A, Panel-B, Panel-C, Panel-D), we generated Kaplan–Meier survival curves grouped by optimized risk score cutoffs. For most of the panels, patients classified as “High Risk” consistently exhibited significantly lower survival probabilities compared to the “Low Risk” group (log-rank *p*-values < 0.01) (Figure S4).

We tested the model’s sensitivity at 95% and 99% specificity levels to understand its clinical utility (Table 5). At 95% specificity, Panel-D showed a sensitivity of 18% for 1-year evaluation. Even at 99% specificity, Panel-A and Panel-D models have 12% sensitivity for a 1-year assessment, potentially identifying a meaningful subset of high-risk patients.

### 3.14. Calibration Curve Analysis

**Figure 7:**
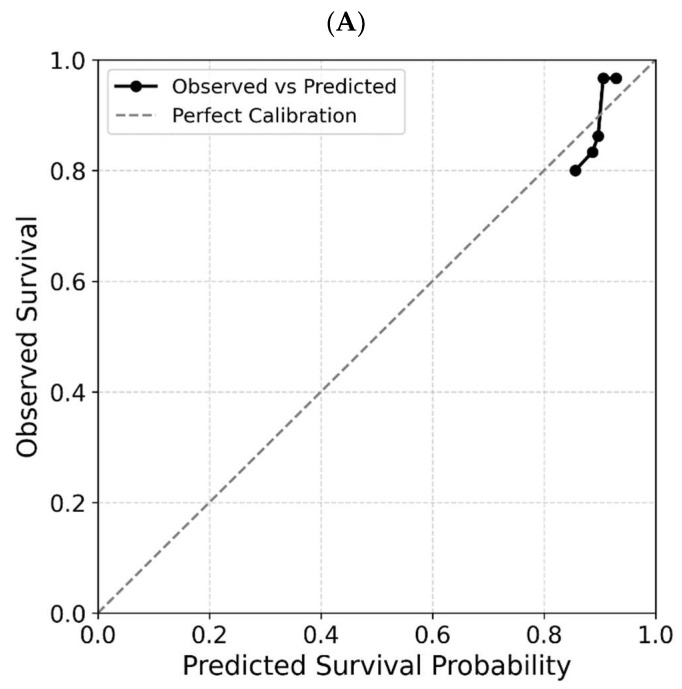

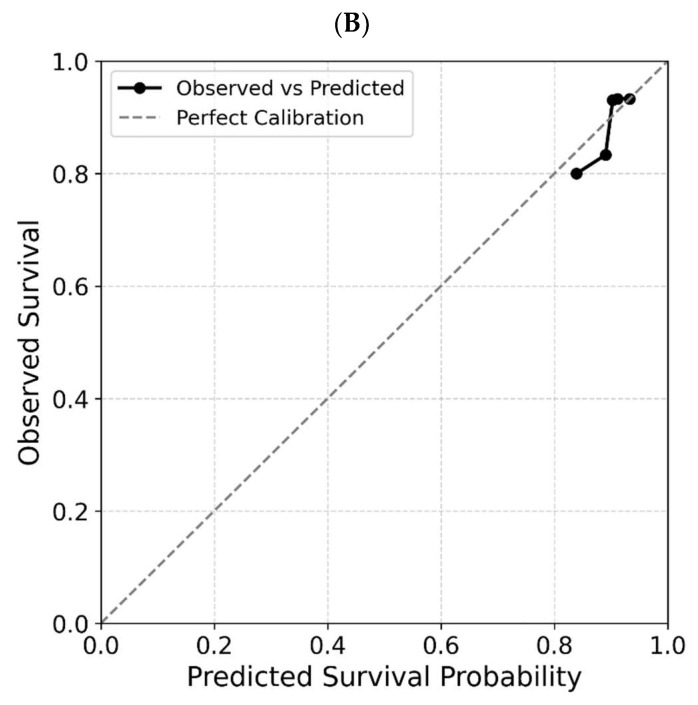
Calibration curves assessing the agreement between predicted and observed survival probabilities for Panel-A (**A**) and Panel-D (**B**) at 1 year for each patient. The Cox model-based predictions showed reasonable calibration, with most panels closely following the diagonal line of perfect calibration for 1-year evaluation points.

Calibration curves were generated for each five-gene panel at 1-, 2-, 3-, and 5-year prediction intervals (Figure 7) to assess the reliability of the model. Each panel demonstrated a reasonably good alignment between the predicted and observed survival rates at the 1-year evaluation time point.

### 3.15. Model Robustness and Discrimination

To assess the stability and reliability of the panel’s estimate, we conducted both bootstrap validation of hazard ratios (HRs) and concordance index (C-index) analyses across the four evaluation time points (1, 2, 3, and 5 years).

In 1000 bootstrap iterations per panel and time point, Panel-D evaluated at one year yielded a mean HR of 8.3 (95% CI: 3.6–19.8 (Table 6). Panel-A evaluated at 1 year yielded a mean HR of 6.1 (95% CI: 3.0–15.3), confirming a strong separation of risk groups. To address the relatively small number of events (17/169, ~10%), we implemented conservative safeguards during bootstrap resampling. Replicates with <5 events overall, <5 patients per group, or <3 events per group were discarded. The C-index is a measure of the model’s ability to rank survival times. Panel-A achieved the highest C-index (0.69) for the 1-year evaluation, and Panel-D showed a C-index above 0.67.

### 3.16. Brier Score Analysis for Prediction Accuracy

The Brier score measures discrimination and calibration. Lower scores indicate better prediction accuracy, with a value of 0.25 representing a non-informative model.

**Figure 8:**
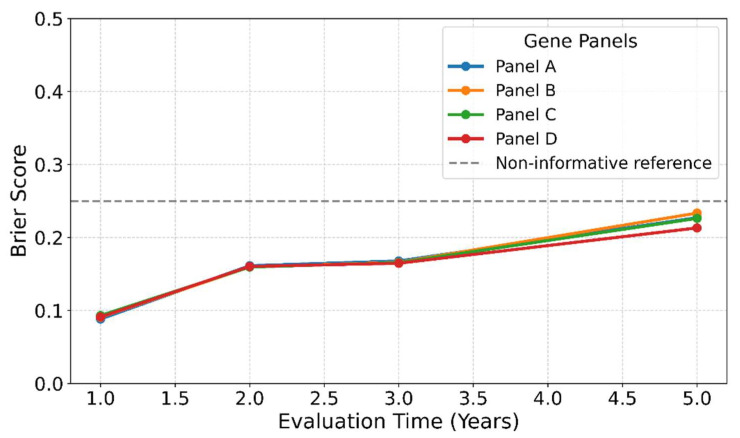
Brier scores at evaluation time points. The Brier score measures the accuracy of survival predictions, where 0 indicates perfect accuracy and 0.25 represents a model that provides no information. All four gene panels showed Brier scores below 0.1 when evaluated for 1-year survival, indicating high predictive accuracy.

Brier scores were less than 0.1 for all four panels at the 1-year evaluation, indicating better prediction accuracy at this time point (Figure 8). These findings complement the model’s high HRs and C-indices, confirming both risk discrimination and probability accuracy.

### 3.17. K-Fold Cross-Validation of Survival Models

We evaluated the performance of each panel using five-fold cross-validation across four evaluation time points: 1, 2, 3, and 5 years. Panel-D assessed at one year yielded the best stratification (HR = 4.61, 95% CI: 1.5–9.7), and its concordance index (C-index) was 0.71, indicating consistent discriminatory performance (Table 7 and Table S4).

## 4. Discussion

Since 1980, the liver cancer death rate has more than doubled. While patients diagnosed at Stage I generally have a relatively favorable prognosis, the outcomes vary widely. About half of Stage I patients live for more than 4 years after they are diagnosed, but a small but significant number of patients die within the first year [8,9] (https://www.cancerresearchuk.org/about-cancer/liver-cancer/survival; https://www.hepb.org/research-and-programs/liver/staging-of-liver-cancer/survival-rates, both accessed on 30 December 2025). Currently, no clinical tests are available to identify which Stage I patients are at a higher risk of early death. Such a panel could potentially inform risk-adapted treatment intensification (e.g., consideration of adjuvant systemic therapy) in a narrowly defined high-risk subgroup.

To address this unmet need, we developed a new multi-gene panel associated with 1-year survival in patients with Stage I liver cancer. We restricted the cohort to Stage I to limit treatment heterogeneity; at this stage, management typically follows the standard of care (SOC), and covariates such as prior treatment are limited. Our results also showed that age and sex have no discriminating potential. This focus reduced potential confounding from variations in therapy, sex, and age in our downstream analyses. It is important to note that while prognostic genes can be identified directly from human data, starting with an animal model and then confirming the results in patients strengthens the findings by integrating two independent systems. Panel-D demonstrated reliable performance throughout internal validation and was particularly associated with 1-year survival in Stage I patients. The Panel-D genes were *ANXA2*, *FXYD3*, *ITGB4*, *PAQR9*, and *USP54*. Panel-A showed reliable performance in most of the internal validation, but failed to meet cross-validation criteria in the current cohort. Evaluation in a larger dataset with greater event accrual may be informative to assess whether performance is affected by limited sample size. The novelty of this work lies in integrating five seemingly weakly correlated genes into a newly constructed biomarker panel. Our approach is mechanism-agnostic, consistent with emerging approaches in molecular diagnostics, where empirical discovery precedes full biological interpretation. For example, the Grail assay uses approximately 100,000 informative differentially methylated regions for multi-cancer early detection (MCED) [35].

This proof-of-concept study underwent rigorous internal validation following the REporting recommendations for tumor MARKer (REMARK) guidelines for prognostic studies [36] and recent guidelines with low event numbers [37]. REMARK guidelines acknowledge that an external validation set “often will not be available” and that “internal” validation procedures such as “cross-validation” and “bootstrapping” are useful for understanding the reliability of the modeling. A split-test approach is unreliable for small event sizes [37,38] and was therefore avoided.

Although external validation is ideal, Stage I liver cancer samples with sufficient events after censoring at the 1-year time point were difficult to obtain. Moreover, RNA-Seq results are affected by various factors, including laboratory conditions, instruments, extraction procedures, sequencing depth, and sample handling [39,40]. Therefore, a publicly available external database may not be appropriate for validating this study. Since we reported results across all five folds (Table S4), each sample was tested exactly once in a distinct validation set, ensuring comprehensive evaluation across the dataset and following the gold-standard REMARK guideline. In clinical settings, an assay is typically developed, locked, and analytically characterized/validated prior to clinical validation. The test and training sets were evaluated using the same locked assay in clinical protocols and in good clinical practice (https://www.accessdata.fda.gov/cdrh_docs/pdf20/P200010S008B.pdf; https://www.accessdata.fda.gov/cdrh_docs/pdf23/P230043B.pdf, both accessed on 30 December 2025). Our panel included only five genes and could be readily adapted to a low-cost qPCR- or ddPCR-based test. Once the test has been adapted and analytically characterized, we plan to validate it using controlled Stage I patient samples with a sufficient number of events.

Recently, a test was shown to be prognostic in patients with clinical Stage I lung adenocarcinoma (LUAD) [41]. Stage I LUAD patients with a positive test had an inferior recurrence-free survival (RFS) of 69% [95% CI: 58–82%] compared to 91% [95% CI: 88–94%], indicating a 1.31-fold enrichment, and a clinical trial has recently been announced (https://grail.com/press-releases/grail-announces-first-patient-tested-with-blood-based-assay-in-global-phase-3-adjuvant-lung-cancer-study/; https://clinicaltrials.gov/study/NCT06564844?cond=Lung%20Adenocarcinoma&spons=AstraZeneca&aggFilters=status:not%20rec&rank=3, both accessed on 30 December 2025). A predictive model was recently published for early-stage HCC (including Stages I and II) to predict 3- and 5-year survival [42]. In contrast, we focused solely on Stage I, where the 4-year survival expectation is high, to identify high-risk patients who are likely to die within 1 year.

One potential limitation of this investigation is the lack of a separate rat model control. To address this limitation, a skilled pathologist examined the sample to ensure accurate identification of both tumor and non-tumor regions. In addition, this study used only three rats for gene expression analysis. To address this concern, we showed that gene expression patterns were consistent across all three animals (Figure 3B). IPA showed the activation of six pathways known to be activated in HCC, validating the biological relevance of our findings. In addition, as a safeguard, the genes with expression changes in the microarray experiment were not used directly in the machine learning models but rather evaluated for their prognostic value using public datasets to limit the likelihood of false discoveries. Similarly, another potential limitation is the use of an FDR threshold of 25%; however, this cutoff is consistent with GSEA guidelines for exploratory analyses, and candidate genes were subsequently validated using TCGA data, reducing the risk of false discovery [16].

Another potential limitation of this study is the relatively small number of events (10%). To address this in bootstrapping, we implemented conservative safeguards during bootstrap resampling. In addition, we applied five-fold cross-validation to evaluate model discrimination and generalization and assessed model calibration using Brier scores and calibration plots. Together, these strategies ensured that hazard ratio estimates and association accuracy metrics provided a reliable evaluation of model performance. We also reported 95% and 80% confidence intervals of the AUC estimates for the multi-gene panels. Some confidence intervals at the early time point with fewer observed events do include 0.5, reflecting statistical uncertainty. We recognize that the modest number of events highlights the importance of future full-scale, multi-year sample collection and validation.

## 5. Conclusions

Although Panel-D underwent rigorous internal validation, the lack of external validation limits the biomarker panel’s clinical significance. To our knowledge, no predictive biomarker panels are currently available in clinics or even in the published literature that identify high-risk patients who are likely to die within 1 year of a Stage I liver cancer diagnosis. However, this proof-of-concept study—if validated in the clinical setting with an external cohort—has the potential to be clinically important.

## 6. Patents

U.S. Provisional Patent Application No. 63/909,244, filed on 31 October 2025, entitled “MULTI-GENE SIGNATURE PREDICTING SURVIVAL IN STAGE I LIVER CANCER,” has been submitted by Rabindra Roy and Ritam Adhikari.

## Figures and Tables

**Table 1:** Stage I liver cancer patient information from TCGA: Clinical and genomic data for Stage I liver cancer patients were downloaded from the TCGA database and censored for downstream analysis. Patients were censored if they were alive before the defined time point but had no follow-up information beyond that point.

Time Point (Year)	Total Number	Patients at Risk	Events Observed	Censored Before Time Point	Median Age (Year)	Total Male	Total Female
1	169	132	17	20	59	119 (70.4%)	50 (29.6%)
2	169	69	31	69	59	119 (70.4%)	50 (29.6%)
3	169	52	32	85	58.5	119 (70.4%)	50 (29.6%)
5	169	23	39	107	58	119 (70.4%)	50 (29.6%)

**Table 2:** (**A**) Prognostic significance of individual genes at 1, 2, 3, and 5 years for Stage I liver cancer: Eleven genes were evaluated for their prognostic capabilities at four different time points (1, 2, 3, and 5 years; see Section 2.8 of the Methods Section) in patients with Stage I liver cancer. Eight of the genes demonstrated significant predictive power (*p*-value < 0.05 and hazard ratio ≠ 1, Table S3) at one or more time points. The top five genes at each time point are highlighted in bold. (**B**) The top five genes at each time point based on AUC from (**A**).

**A**				
**Gene**	**1 Year Time Point AUC**	**2 Year Time Point AUC**	**3 Year Time Point AUC**	**5 Year Time Point AUC**
*ANXA2*	**0.61**	**0.60**	**0.59**	**0.63**
*AOC1*	**0.60**	**0.57**	**0.58**	0.54
*CCL21*	0.35	0.42	0.44	0.48
*CD24*	0.39	0.57	**0.58**	0.57
*CXCL6*	0.45	0.47	0.45	0.48
*FXYD3*	0.57	0.47	0.56	**0.61**
*GK*	**0.66**	**0.57**	0.57	0.48
*ITGB4*	0.37	**0.57**	**0.59**	**0.60**
*MGP*	0.34	0.40	0.39	0.35
*PAQR9*	**0.62**	0.55	0.56	**0.59**
*USP54*	**0.63**	**0.61**	**0.62**	**0.57**
**B**				
**Panel Name**		**Gene Name**		
Panel-A		*ANXA2*, *AOC1*, *GK*, *PAQR9*, *USP54*		
Panel-B		*ANXA2*, *AOC1*, *GK*, *ITGB4*, *USP54*		
Panel-C		*ANXA2*, *AOC1*, *CD24*, *ITGB4*, *USP54*		
Panel-D		*ANXA2*, *PAQR9*, *FXYD3*, *ITGB4*, *USP54*		

**Table 3:** LASSO-regularized regression for risk score construction: LASSO did not identify any individual genes in the panel as contributing a unique predictive value to the current modeling framework. However, the aggregate signal across genes, as reflected in AUC values greater than 0.65, suggests potentially useful prognostic information, especially for Stage I.

Gene Panel	AUC	Evaluation Year
Panel-D	0.674	5 year
Panel-D	0.673	1 year
Panel-A	0.652	1 year
Panel-C	0.651	5 year

**Table 4:** The AUC values with 80% and 95% CIs for each multi-gene panel at each time point.

Panel	Eval Year	AUC	CI95 Lower	CI95 Upper	CI80 Lower	CI80 Upper
Panel-A	1	0.70	0.41	0.75	0.50	0.74
Panel-A	2	0.63	0.49	0.63	0.53	0.62
Panel-A	3	0.65	0.49	0.65	0.54	0.64
Panel-A	5	0.57	0.43	0.67	0.47	0.64
Panel-B	1	0.66	0.43	0.73	0.52	0.70
Panel-B	2	0.67	0.52	0.67	0.56	0.66
Panel-B	3	0.68	0.53	0.70	0.57	0.68
Panel-B	5	0.58	0.44	0.64	0.48	0.62
Panel-C	1	0.64	0.43	0.70	0.50	0.67
Panel-C	2	0.66	0.53	0.67	0.57	0.66
Panel-C	3	0.68	0.54	0.70	0.57	0.69
Panel-C	5	0.61	0.49	0.63	0.52	0.62
Panel-D	1	0.64	0.49	0.68	0.55	0.67
Panel-D	2	0.65	0.53	0.67	0.57	0.65
Panel-D	3	0.67	0.54	0.70	0.58	0.68
Panel-D	5	0.67	0.56	0.71	0.60	0.70

**Table 5:** One-year survival probability predicted by the multivariate Cox model: Although patients with Stage I liver cancer patients generally have a favorable prognosis, a small but significant fraction die within the first year. Two of our gene panels were able to identify approximately 12% of high-risk patients, with an exceptionally high specificity of 99%.

Panel	Eval Year	Sensitivity at 95% Specificity	Sensitivity at 99% Specificity
Panel-A	1	0.12	0.12
Panel-A	2	0.06	0.06
Panel-A	3	0.06	0.06
Panel-A	5	0.05	0.05
Panel-B	1	0.18	0
Panel-B	2	0.16	0
Panel-B	3	0.16	0
Panel-B	5	0.21	0
Panel-C	1	0.18	0
Panel-C	2	0.16	0
Panel-C	3	0.16	0
Panel-C	5	0.15	0
Panel-D	1	0.18	0.12
Panel-D	2	0.13	0.06
Panel-D	3	0.12	0.06
Panel-D	5	0.13	0.05

**Table 6:** Bootstrap validation and C-index: To assess the robustness of the risk group stratification, we performed 1000 bootstrap resamples for the four gene panels’ survival predictions at different time points. Across resamples, the 95% confidence intervals of HRs ranged from 3.0 to 15.4 at 1 year for the Panel-A’s prediction of survival probability. The Panel-D performed even better in predicting higher risk (HR = 8.3) for 1-year survival, with a 95% confidence interval of 3.6–19.8.

Panel	Eval Year	HR Mean	CI Lower	CI Upper
Panel-A	1	6.13	2.95	15.33
Panel-A	2	3.71	2.01	7.77
Panel-A	3	3.57	1.99	6.79
Panel-A	5	3.00	1.69	6.08
Panel-B	1	6.78	3.05	16.49
Panel-B	2	4.22	2.37	8.13
Panel-B	3	4.25	2.40	8.32
Panel-B	5	3.57	2.09	6.76
Panel-C	1	7.18	3.22	17.60
Panel-C	2	4.61	2.51	10.01
Panel-C	3	4.68	2.60	10.09
Panel-C	5	3.79	2.21	7.28
Panel-D	1	8.23	3.60	19.77
Panel-D	2	4.55	2.62	9.26
Panel-D	3	4.50	2.40	9.48
Panel-D	5	3.73	2.04	7.30

**Table 7:** K-fold cross-validation suggests generalization of the model predicting 1-year survival: We examined the accuracy of the gene panels for predicting survival at one year. We used 5-fold cross-validated Cox proportional hazards modeling with an optimized risk score threshold. The best stratification and its C-index of panel and evaluation year are highlighted in bold.

Panel	Eval Year	Folds	Mean HR	Median HR	HR CI Lower	HR CI Upper	Mean C-Index	C-Index_Std	N Folds Used
Panel-A	2	5	0.79	0.71	0.35	1.37	0.50	0.10	4
Panel-A	3	5	1.45	1.10	0.73	2.91	0.56	0.07	5
Panel-A	5	5	0.72	0.75	0.42	0.96	0.43	0.08	5
Panel-B	1	5	1.96	1.75	0.89	2.94	0.66	0.09	5
Panel-B	2	5	1.51	1.64	0.49	2.32	0.55	0.08	4
Panel-B	3	5	1.76	1.44	0.90	3.16	0.54	0.05	4
Panel-B	5	5	1.11	0.62	0.42	2.69	0.48	0.11	5
Panel-C	1	5	3.68	2.33	1.22	8.42	0.66	0.09	4
Panel-C	2	5	2.78	2.29	1.55	4.84	0.60	0.10	4
Panel-C	3	5	2.07	1.83	0.76	3.78	0.57	0.10	4
Panel-C	5	5	1.03	0.79	0.44	2.09	0.53	0.08	5
Panel-D	**1**	**5**	**4.61**	**3.74**	**1.47**	**9.72**	**0.71**	**0.07**	**5**
Panel-D	2	5	1.62	1.39	0.42	3.22	0.56	0.10	4
Panel-D	3	5	1.54	1.14	0.56	2.80	0.57	0.10	5
Panel-D	5	5	1.21	0.73	0.48	2.83	0.54	0.11	5

## Data Availability

The datasets generated in this study are available from the corresponding author upon request. The microarray data generated in this study have been deposited in the NCBI’s Gene Expression Omnibus (GEO) under accession number GSE309883. The TCGA data used in this study are publicly available. The custom scripts used for data analysis in this study are available on a GitHub repository at the following link: https://github.com/TechnoKirb/GeorgetownResearch, accessed on 30 December 2025.

## Supplementary Materials

The following supporting information can be downloaded at https://www.mdpi.com/article/10.3390/cimb48020136/s1.

## Abbreviations

LEC	Long–Evans Cinnamon
HCC	Hepatocellular Carcinoma
WD	Wilson’s Disease
MASLD	Metabolic Dysfunction-Associated Liver Disease
SOC	Standard of Care
TCGA	The Cancer Genome Atlas
RVM	Random Variance Model
GSEA	Gene Set Enrichment Analysis
WCSS	Within-Cluster Sum of Squares
IPA	Ingenuity Pathway Analysis
HPA	Human Protein Atlas
KM	Kaplan–Meier
AUC	Area Under the Curve
IPCW	Inverse-Probability-of-Censoring Weighted
HR	Hazard Ratio
ROC	Receiver Operating Characteristic
C-Index	Concordance Index
CI	Confidence Interval
