# Supplementary material for: A Multi-Gene Signature Associated with 1-Year Survival in Patients with Stage I Liver Cancer: Integration of Preclinical and TCGA Data
Source: Curr Issues Mol Biol. 2026 Jan 27;48(2):136. doi: 10.3390/cimb48020136 (PMC12939873; doi:10.3390/cimb48020136)
Supplement: Supplementary file 1 [file cimb-48-00136-s001.zip › cimb-4103780-supplementary.pdf]

Figure S1: Agglomerative Hierarchical Clustering. This hierarchical clustering organizes genes based on expression similarities, highlighting key clusters for pathway analysis and suggest two possible clusters within both the upregulated and downregulated genes.
We then tested further K-mean clustering analysis. Dendrograms → 1A: Upregulated. 1B: downregulated

A

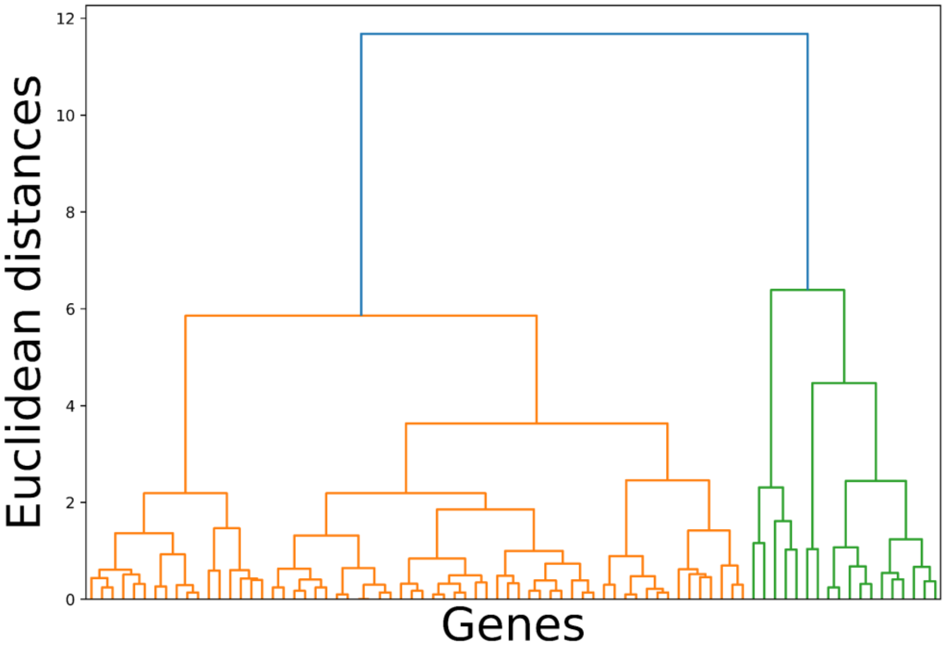

B

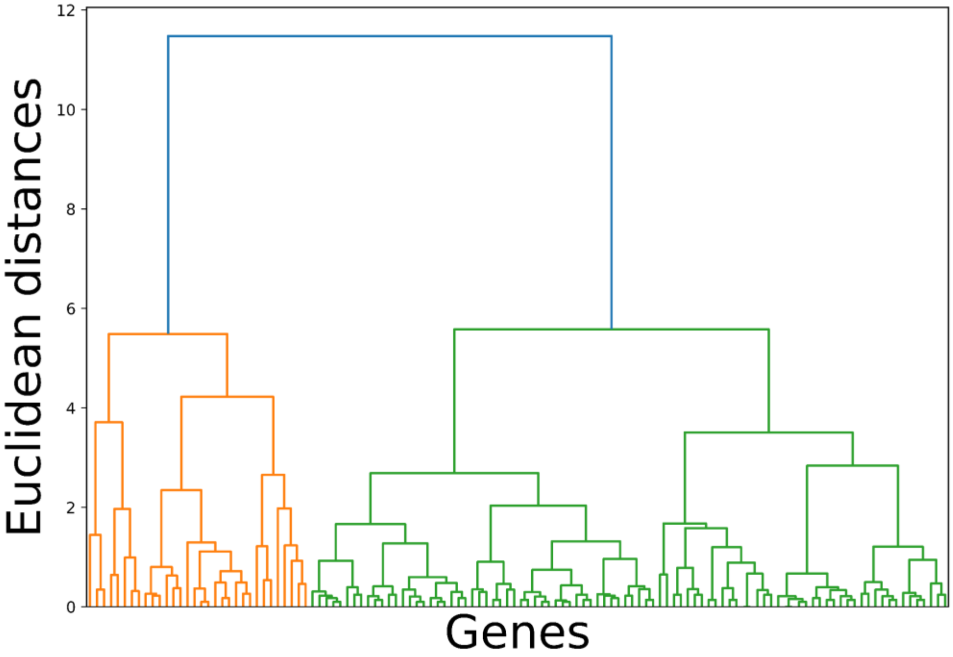

Figure S2: IPA to discover/validate pathways. Interaction of different pathways within upregulated cluster 2 (A). We discovered six tumor-activated pathways shown for the first time in WD-mediated HCC, opening new avenues for prognostics and therapeutic research (B).

A

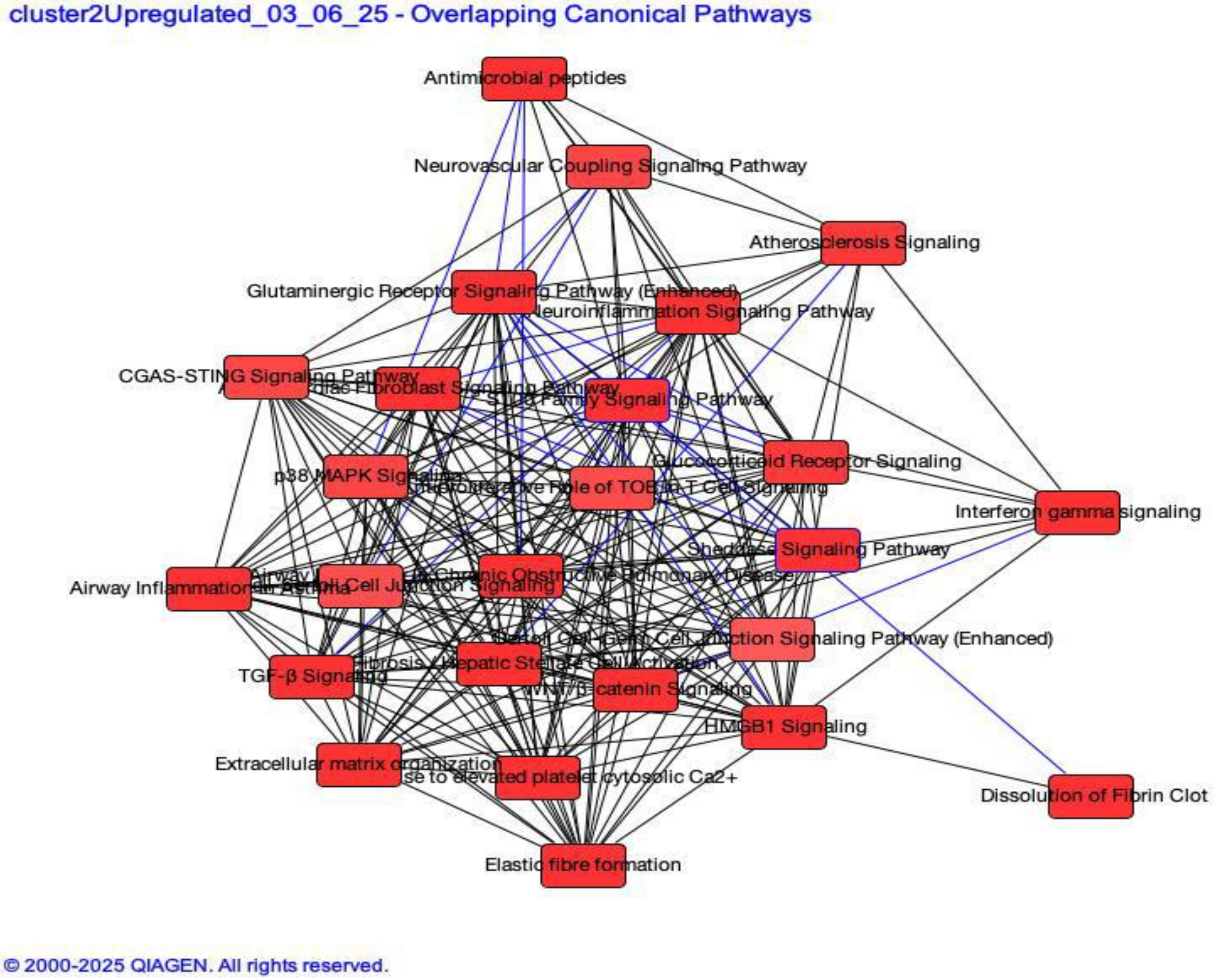

B

Pathway Name	Z-score	-log10 (p-value)	Previous Reference
S100 Family Signaling	2.33	3.600	Not WD, yes HCC
Extracellular Matrix Organization	2	3.391	Not WD, yes HCC
Response to Elevated platelet cytosolic Ca2+	2	3.026	Not WD, yes HCC
HMGB1 Signaling	2.00	2.801	Not WD, yes HCC
Sertoli Cell-Sertoli Cell Junction Signaling	2.236	1.757	Not WD, yes HCC
Cachexia	2.000	1.583	Not WD, yes HCC

Table S1. Top 10 genes from each cluster

Upregulated cluster 1						Downregulated cluster 1				
**Gene symbol**	**1(N/T)**	**2(N/T)**	**3(N/T)**	**Fold Change**		**Gene symbol**	**1(N/T)**	**2(N/T)**	**3(N/T)**	**Fold Change**
Gpx2	-1	-3.9	-3.2	6.5		*Car3*	1.6	3.1	2.1	-4.9
Cxcl6	-1.3	-3.2	-3.1	5.8		*Pcp4l1*	3	2.1	1.1	-4.2
Msln	-0.7	-3.6	-2.4	4.7		*Tpd52l1*	0.9	1.6	2.1	-2.9
Mgp	-0.7	-3.7	-2.2	4.6		*Gk*	1.5	1.5	1.6	-2.9
Itgb4	-2.3	-2	-2.4	4.6		*Elovl2*	1.4	1.6	1.4	-2.8
Pdpn	-1.2	-3	-2	4.2		*Paqr9*	0.8	1.7	1.9	-2.8
Abp1	-0.5	-3.4	-2.3	4.2		*Fabp12*	1.8	1.4	1.2	-2.8
Usp54	-3.2	-0.4	-2.6	4.2		*Car3*	1.3	1.7	1.4	-2.8
Tnnt2	-1.3	-2.6	-2.1	4.1		*Lrtm2*	1	1.6	1.9	-2.8
Nupr1	-1.4	-1.8	-2.9	4.1		*Pdzk1*	0.9	2.1	1.5	-2.8

Upregulated cluster 2						Downregulated cluster 2				
**Gene symbol**	**1(N/T)**	**2(N/T)**	**3(N/T)**	**Fold Change**		**Gene symbol**	**1(N/T)**	**2(N/T)**	**3(N/T)**	**Fold Change**
Ankrd1	-1.3	-2.7	-1.7	3.8		*Cyp2c*	2.2	2.3	2.8	-5.4
Ctgf	-0.8	-2.8	-1.5	3.3		*Cyp1a2*	1.7	3.2	2.2	-5.2
Igfbp5	-1.5	-2.1	-1.6	3.3		*Car3*	1.6	3.1	2.1	-4.9
Fxyd3	-0.6	-2.8	-1.7	3.2		*Nudt11*	1.7	3.3	1.5	-4.4
Ccl21b	-1.1	-1.9	-2	3.2		*Sez6*	1.5	2.1	2.7	-4.3
Lcn2	-2	-0.7	-2.3	3.2		*Obp3*	2.4	1.4	2.6	-4.3
Klf5	-0.8	-2.3	-1.9	3.2		*Slc22a8*	2.4	1.8	2	-4.2
Cd44	-1.4	-2.1	-1.4	3.1		*Pcp4l1*	3	2.1	1.1	-4.2
Cd24	-1.7	-1.6	-1.4	3		*LOC684425*	2	1.9	2	-4
Anxa2	-1	-2.1	-1.6	3		*RGD1565709*	1.2	3.3	1.5	-4

Table S2: Survival-associated genes. We selected the top ten genes in each cluster. Web-based tools like HPA, OncoLnc, and KM plotter were used to measure survival probability using TCGA-Liver Cancer data to understand the relevance of human liver cancer.
Although many of these genes have been shown as individual potential prognostic markers in liver cancer, their interactions and combined influence on survival have not been explored.
All 11 genes detected from the TCGA database were previously reported as possible biomarkers for liver cancer by 11 different groups.

**Human Gene**	**COX Coefficient (OncoLnc)**	**5 year Survival Probability High Expression (%) (HPA)**	**5 year Survival Probability Low Expression (%) (HPA)**	**Hazard Ratio (KM plotter)**
CCL21	-0.162	51	40	0.68
MGP	-0.129	52	42	0.59
GK	-0.098	55	38	0.66
ITGB4	-0.06	30	54	0.63
PAQR9	0.136	41	55	1.63
CXCL6	0.177	42	66	2.02
AOC1	0.192	38	54	1.52
FXYD3	0.194	38	60	1.69
USP54	0.2	42	54	1.7
ANXA2	0.261	39	56	2.1
CD24	0.289	36	53	1.61

Table S3: Prognostic significance of individual genes at 1-, 2-, 3-, and 5-year survival for Stage I liver cancer

Gene	HR at 1 year	95% CI at 1 year	HR at 2 year	95% CI at 2 year	HR at 3 year	95% at CI 3 year	HR at 5 year	95% CI at 5 year
GK	3.864	1.49–10.02	2.163	1.05–4.46	2.05	1.00–4.20	1.472	0.78–2.78
ANXA2	2.62	1.01–6.79	2.223	1.08–4.58	2.338	1.14–4.78	2.015	1.07–3.78
AOC1	2.305	0.88–6.06	1.796	0.89–3.65	1.707	0.85–3.43	1.593	0.82–3.10
CCL21	0.117	0.01–0.88	0.417	0.20–0.87	0.443	0.21–0.92	0.534	0.27–1.05
CD24	0.402	0.14–1.14	1.956	0.92–4.15	2.141	1.03–4.44	1.857	0.94–3.67
CXCL6	0.642	0.24–1.69	0.656	0.32–1.34	0.626	0.31–1.27	0.716	0.38–1.35
FXYD3	1.574	0.60–4.14	0.763	0.36–1.62	1.441	0.69–2.99	1.567	0.83–2.95
ITGB4	0.367	0.13–1.04	1.861	0.89–3.88	2.039	1.00–4.17	1.767	0.91–3.44
MGP	0.267	0.10–0.69	0.39	0.19–0.82	0.404	0.20–0.84	0.387	0.19–0.78
PAQR9	3.179	1.21–8.36	1.727	0.66–4.50	1.795	0.69–4.66	1.628	0.75–3.55
USP54	2.712	1.03–7.13	2.18	1.08–4.41	2.056	1.03–4.12	1.531	0.82–2.87

Figure S3 Kaplan-Meier plots for individual genes

AOC1 – Kaplan-Meier Survival by Time Point

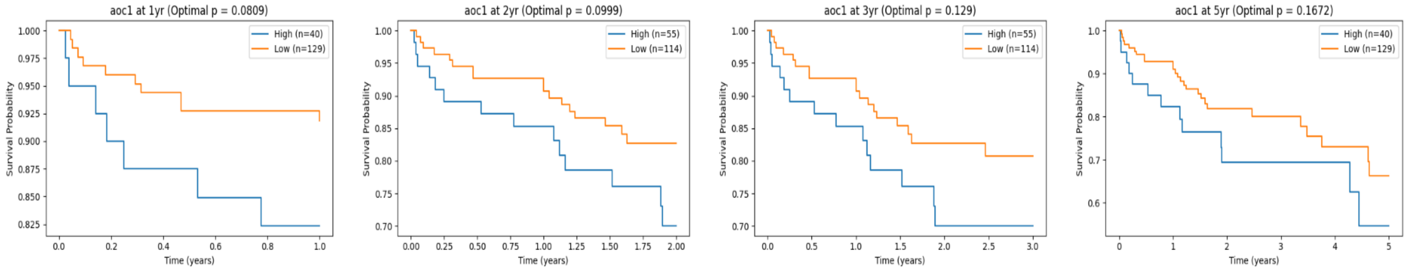

CCL21 – Kaplan-Meier Survival by Time Point

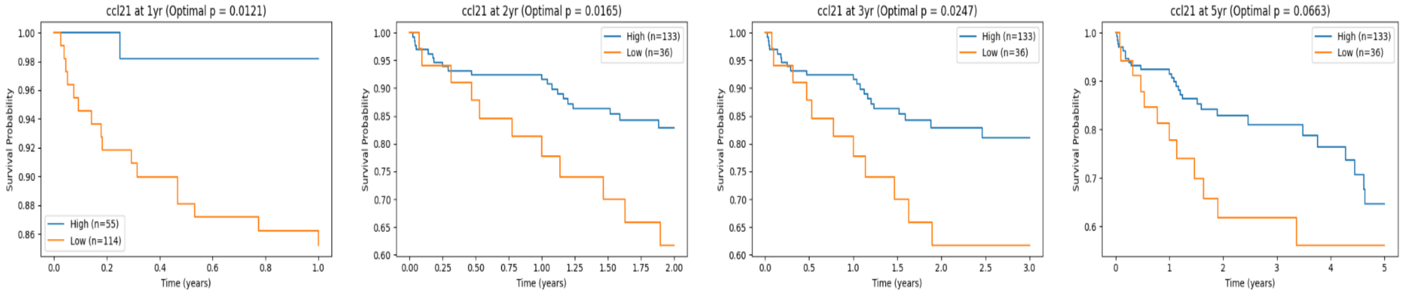

CD24 – Kaplan-Meier Survival by Time Point

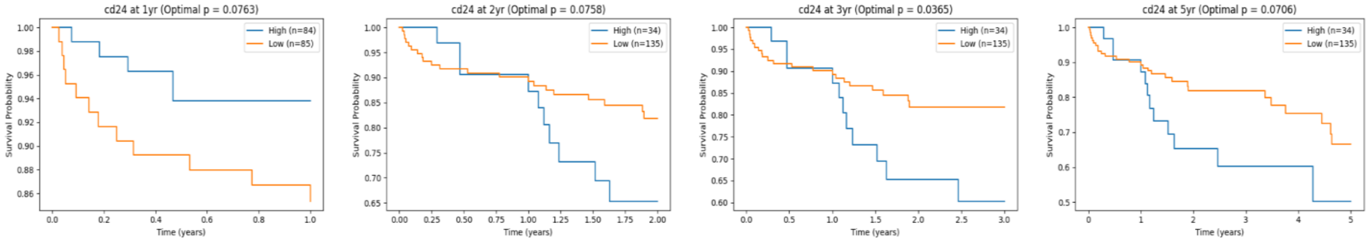

CXCL6 – Kaplan-Meier Survival by Time Point

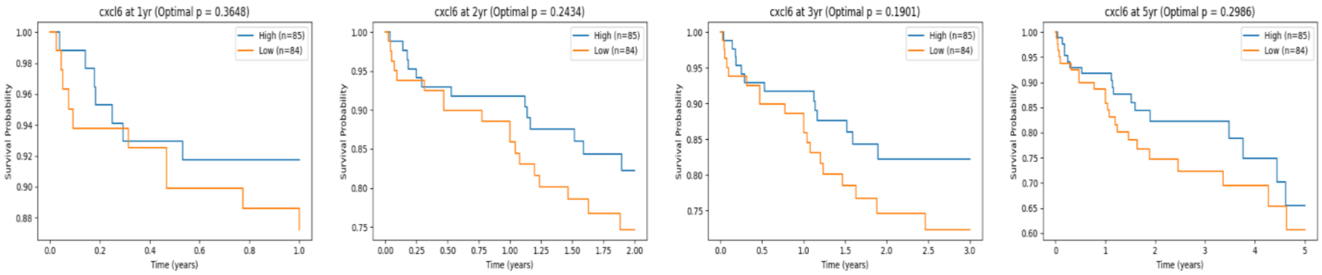

FXYD3 – Kaplan-Meier Survival by Time Point

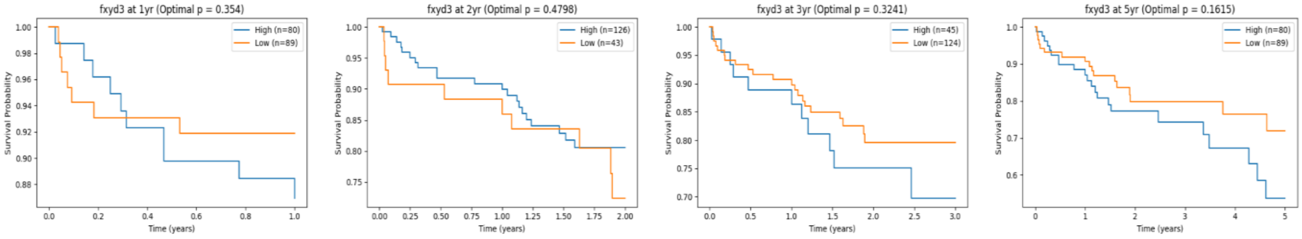

GK – Kaplan-Meier Survival by Time Point

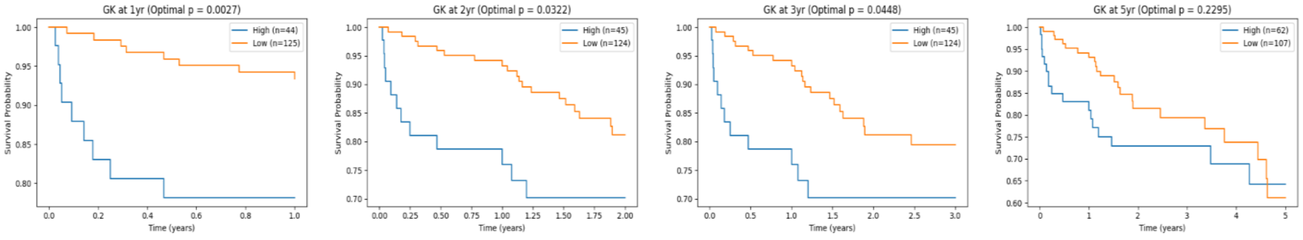

ITGB4 – Kaplan-Meier Survival by Time Point

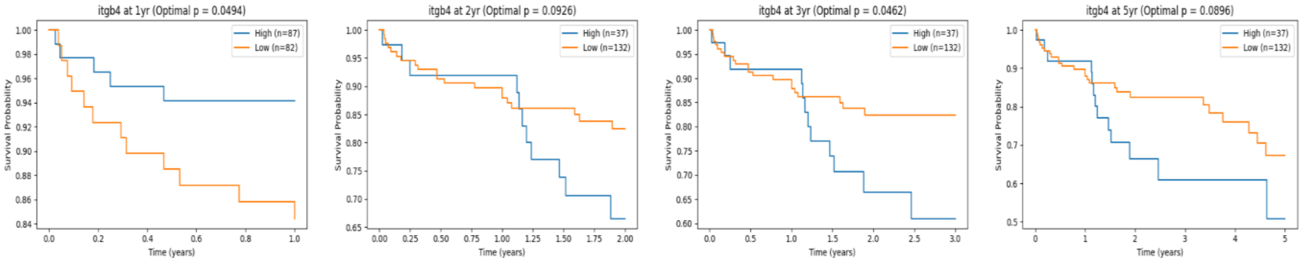

MGP – Kaplan-Meier Survival by Time Point

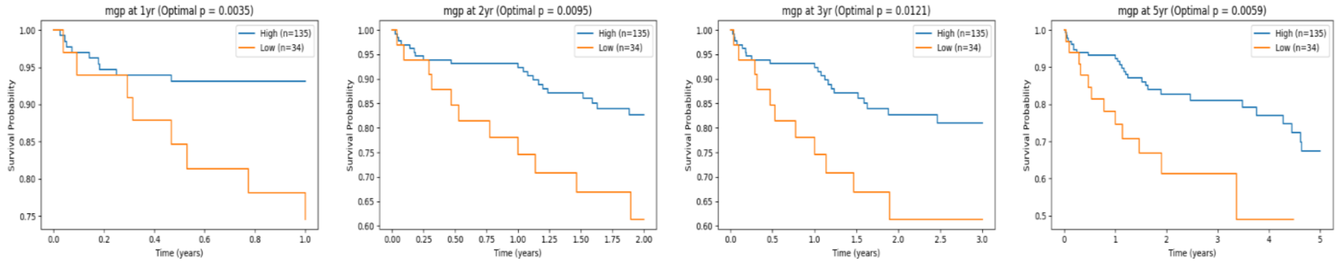

PAQR9 – Kaplan-Meier Survival by Time Point

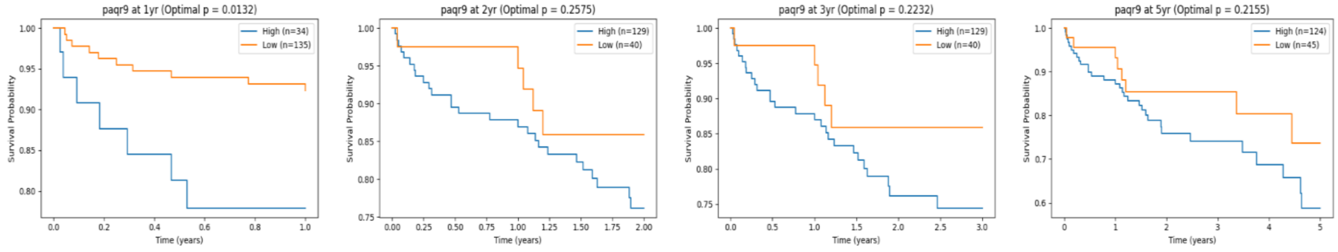

USP54 – Kaplan-Meier Survival by Time Point

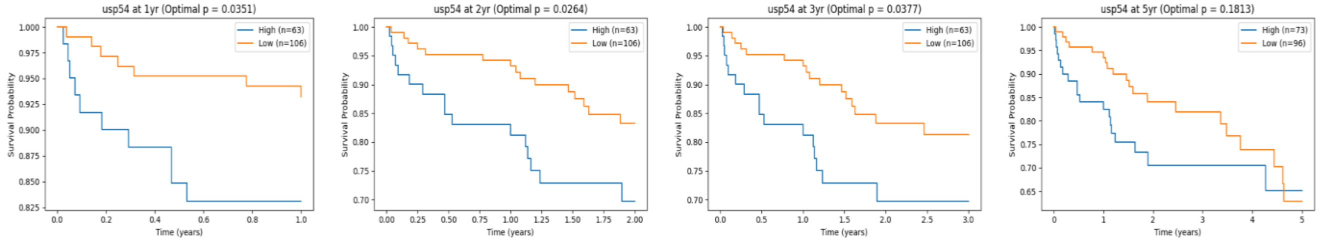

ANXA2 – Kaplan-Meier Survival by Time Point

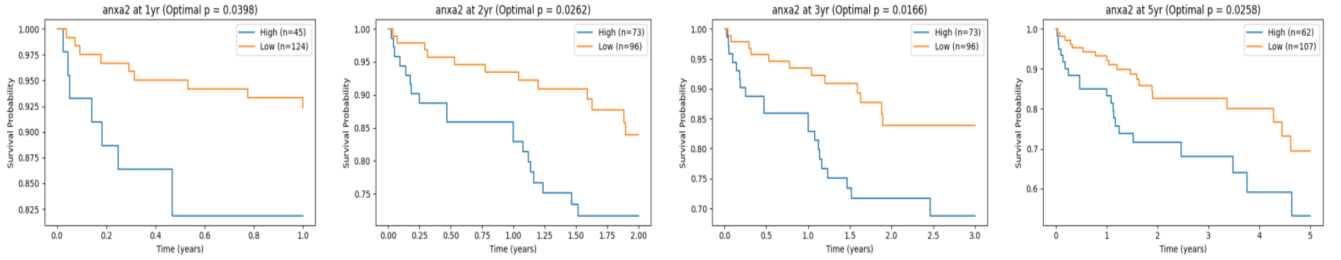

Figure S4. Kaplan-Meier Survival curve with four panels. Patient-level risk scores were computed from multivariate Cox proportional hazards time-dependent AUC models and used to define high- vs low-risk groups based on optimal risk score thresholds using log-rank test–based minimization of the p-value. Kaplan–Meier plots demonstrated clear and consistent survival separation between risk groups across many panel–year combinations ($p < 0.05$).

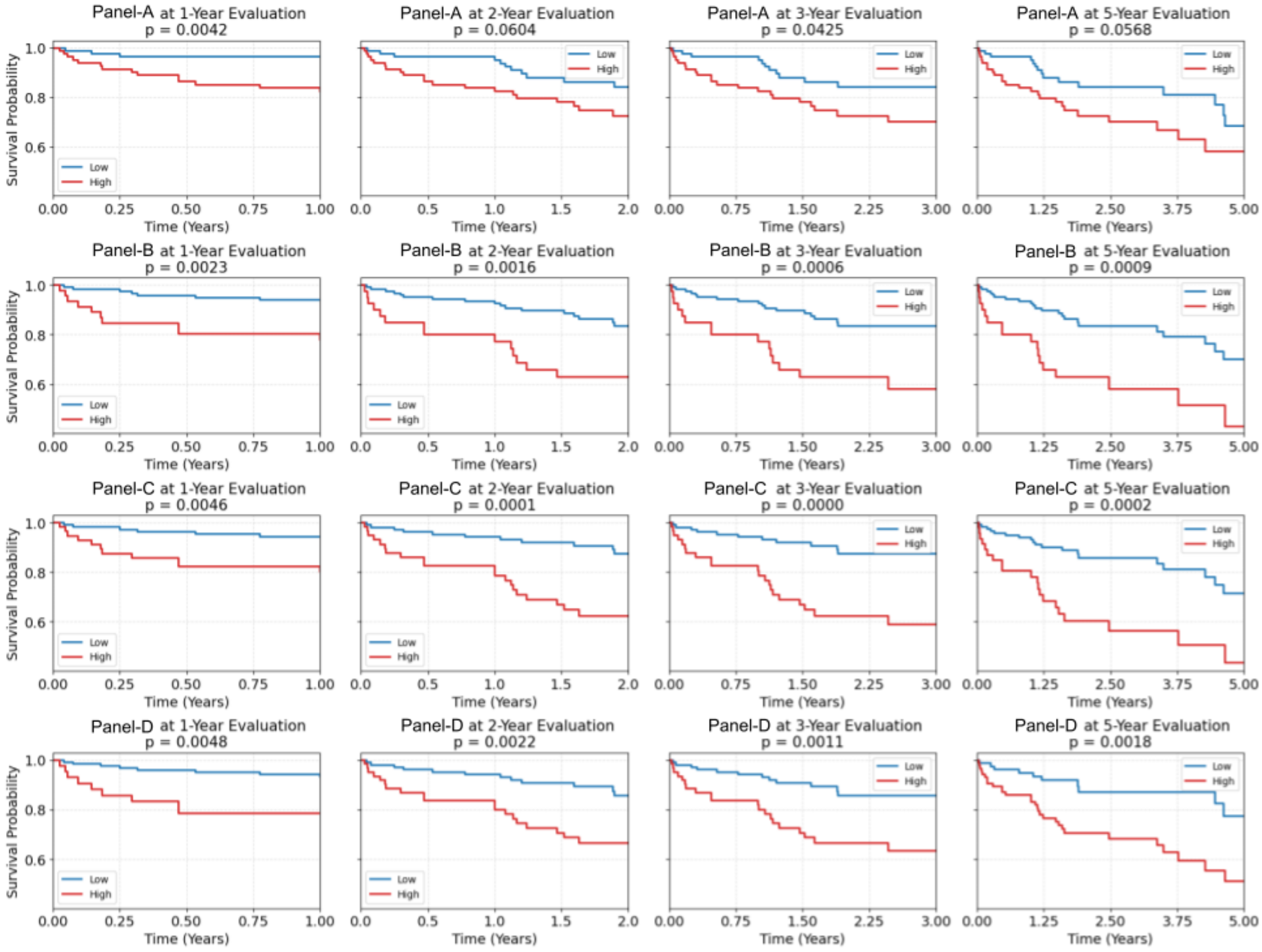

Table S4: Per-fold results: Individual fold results for 5-fold CV suggest that the Panel-D is best for 1-year risk prediction

Panel	Eval_Year	Fold	HR	C_index	Risk_Cutoff_RR	Cutoff_Percentile	Events_in_Test	N_in_Test
Panel-A	1	1	0	0.512	2.677	76	3	34
Panel-A	1	2	18726836.08	0.819	1.322	41	3	34
Panel-A	1	3	2.737	0.803	1.032	35	4	34
Panel-A	1	4	16861948.33	0.647	1.344	48	4	34
Panel-A	1	5	0	0.473	2.099	80	3	33
Panel-A	2	1	0.325	0.341	1.086	44	6	34
Panel-A	2	2	0.627	0.503	1.639	74	6	34
Panel-A	2	3	1.411	0.539	1.192	35	6	34
Panel-A	2	4	0.789	0.607	1.106	33	7	34
Panel-A	2	5	0	0.338	2.112	56	6	33
Panel-A	3	1	0.785	0.521	0.934	35	6	34
Panel-A	3	2	1.556	0.631	1.058	21	6	34
Panel-A	3	3	3.061	0.654	1.152	25	7	34
Panel-A	3	4	0.724	0.473	1.368	41	7	34
Panel-A	3	5	1.098	0.506	1.239	29	6	33
Panel-A	5	1	0.656	0.451	1.013	26	8	34
Panel-A	5	2	0.978	0.538	1.412	48	8	34
Panel-A	5	3	0.832	0.433	1.118	44	8	34
Panel-A	5	4	0.389	0.293	0.666	25	8	34
Panel-A	5	5	0.751	0.42	1.061	46	7	33
Panel-B	1	1	1.745	0.683	1.969	76	3	34
Panel-B	1	2	1.707	0.628	2.245	76	3	34
Panel-B	1	3	2.56	0.787	1.106	48	4	34
Panel-B	1	4	2.983	0.689	1.894	71	4	34
Panel-B	1	5	0.797	0.505	1.087	49	3	33

Panel-B	2	1	1.364	0.482	1.307	75	6	34
Panel-B	2	2	0.419	0.497	1.515	64	6	34
Panel-B	2	3	1.911	0.675	1.136	26	6	34
Panel-B	2	4	2.348	0.562	1.417	68	7	34
Panel-B	2	5	0	0.391	3.758	79	6	33
Panel-B	3	1	3.279	0.544	1.276	80	6	34
Panel-B	3	2	15730698.57	0.875	1.086	30	6	34
Panel-B	3	3	1.207	0.599	2.274	74	7	34
Panel-B	3	4	0.879	0.473	2.826	80	7	34
Panel-B	3	5	1.671	0.526	1.496	60	6	33
Panel-B	5	1	0.399	0.484	1.72	74	8	34
Panel-B	5	2	1.088	0.565	1.944	80	8	34
Panel-B	5	3	0.566	0.351	1.749	80	8	34
Panel-B	5	4	0.616	0.385	0.967	43	8	34
Panel-B	5	5	2.862	0.634	1.23	73	7	33
Panel-C	1	1	1.185	0.598	2.083	80	3	34
Panel-C	1	2	0	0.628	1.91	80	3	34
Panel-C	1	3	8.857	0.787	1.469	80	4	34
Panel-C	1	4	2.983	0.714	1.072	57	4	34
Panel-C	1	5	1.684	0.548	1.228	70	3	33
Panel-C	2	1	1.726	0.441	1.087	71	6	34
Panel-C	2	2	2.854	0.606	1.506	58	6	34
Panel-C	2	3	1.531	0.701	1.38	64	6	34
Panel-C	2	4	5.005	0.652	1.235	59	7	34
Panel-C	2	5	0	0.457	2.839	79	6	33
Panel-C	3	1	2.479	0.592	1.156	76	6	34
Panel-C	3	2	0	0.625	1.327	73	6	34
Panel-C	3	3	3.886	0.698	2.055	71	7	34
Panel-C	3	4	0.728	0.432	1.372	54	7	34

Panel-C	3	5	1.175	0.558	1.447	63	6	33
Panel-C	5	1	0.79	0.497	1.307	54	8	34
Panel-C	5	2	0.984	0.581	1.62	80	8	34
Panel-C	5	3	0.403	0.394	1.393	79	8	34
Panel-C	5	4	2.212	0.606	1.968	80	8	34
Panel-C	5	5	0.78	0.565	0.961	44	7	33
Panel-D	1	1	10.125	0.793	1.597	80	3	34
Panel-D	1	2	1.441	0.745	1.627	79	3	34
Panel-D	1	3	1.68	0.656	0.712	56	4	34
Panel-D	1	4	3.741	0.748	0.837	51	4	34
Panel-D	1	5	6.042	0.602	0.87	64	3	33
Panel-D	2	1	3.37	0.582	1.195	79	6	34
Panel-D	2	2	1.356	0.594	1.393	56	6	34
Panel-D	2	3	1.427	0.656	1.242	79	6	34
Panel-D	2	4	0.345	0.388	0.753	43	7	34
Panel-D	2	5	0	0.464	2.672	80	6	33
Panel-D	3	1	2.018	0.651	1.122	60	6	34
Panel-D	3	2	1.138	0.476	1.2	68	6	34
Panel-D	3	3	2.885	0.728	1.852	78	7	34
Panel-D	3	4	1.141	0.473	1.279	58	7	34
Panel-D	3	5	0.496	0.526	1.411	71	6	33
Panel-D	5	1	0.727	0.516	1.376	71	8	34
Panel-D	5	2	0.56	0.366	1.564	75	8	34
Panel-D	5	3	0.474	0.495	1.482	79	8	34
Panel-D	5	4	2.999	0.644	1.238	48	8	34
Panel-D	5	5	1.301	0.679	1.122	49	7	33
